# Phenotypic characterization of extended-spectrum-beta-lactamase producing *E. coli* from healthy individuals, patients, sewage sludge, cattle, chickens and raw meat

**DOI:** 10.12669/pjms.334.12647

**Published:** 2017

**Authors:** Rabia Saleem, Hasan Ejaz, Aizza Zafar, Sonia Younas, Ahsan Waheed Rathore

**Affiliations:** 1 Rabia Saleem, M.Phil Scholar, Department of Microbiology, The Children’s Hospital and The Institute of Child Health, Lahore, Pakistan; 2 Hasan Ejaz, M.Phil, PhD. Department of Microbiology, The Children’s Hospital and The Institute of Child Health, Lahore, Pakistan; 3 Aizza Zafar, M.Phil. Department of Microbiology, The Children’s Hospital and The Institute of Child Health, Lahore, Pakistan; 4 Sonia Younas, M.Phil Scholar, Department of Microbiology, Al-Razi HealthCare, Lahore, Pakistan; 5 Ahsan Waheed Rathore, MRCPCH, FRCP, Department of Paediatric Medicine, The Children’s Hospital and The Institute of Child Health, Lahore, Pakistan

**Keywords:** ESBL-*E. coli*, Human and non-human sources, Antimicrobial resistance

## Abstract

**Objective:**

The present study aimed to determine the frequency and antimicrobial profile of ESBL-producing isolates of *E. coli* in different environments.

**Methods:**

This cross-sectional study was conducted at The Children’s Hospital and The Institute of Child Health, Lahore from July to December 2015. The faecal specimens from healthy individuals, patients, sewage sludge, cattle, chickens and raw meat (n = 122) were processed for microbiological analysis using MacConkey agar supplemented with cefotaxime. The identification of organisms was confirmed by API 10S and antimicrobial resistance profile was recorded by Kirby-Bauer disc diffusion method.

**Results:**

On the basis of screening, 77 (63.0%) specimens were found to be positive for ESBL production. The confirmation of 74 (60.0%) ESBL producing *E. coli* was done using double disc synergy test (DDST). The frequency of ESBL producing *E. coli* was found to be 17 (57.0%) in healthy individuals, 15 (53.0%) in patients, 10 (66.0%) in cattle faeces, 5 (71.0%) in sewage sludge, 14 (70.0%) in raw meat and 13 (59.0%) in chicken faeces. All of these isolates were resistant to cephalosporins and some of these were resistant to fluoroquinolones and meropenem. None of the isolates showed resistance to cefoperazone-sulbactam, imipenem, piperacillin-tazobactam and amikacin.

**Conclusion:**

The prevalence of ESBL-producing *E. coli* was recorded in all the environments, suggesting a global expansion of these enzymes.

## INTRODUCTION

*Escherichia coli* (*E. coli*) is a Gram negative rod-shaped bacterium that commonly exists in the intestine of human and other warm-blooded organisms. Most strains of *E. coli* are not supposed to cause any harm but some serotypes of *E. coli* are harmful and can cause pathology.^1^ Virulent strains of *E. coli* can cause various diseases in humans and animals. In humans it can cause gastroenteritis, urinary tract infections, food poisoning, haemolytic-uremic syndrome, peritonitis mastitis, septicaemia, pneumonia, diarrhoea and neonatal meningitis.^2^ The main passage of transmission of *E. coli* infections is faecal-oral route. During slaughter and carcass processing, the shedding of *E. coli* could prove to be the important source of contamination.^3^ It can also be transmitted by food and travelling.^4^ The humans colonised with ESBL-producing *E. coli* can release large quantities of bacteria into the environment which will then enter the transmission cycle through sewage, water, and soil.^5^ ESBL-producing *E. coli* are resistant to penicillins, cephalosporins but are susceptible to cephamycins and carbapenems.^6^

High levels of resistance among the clinical isolates have come up to the alarming situation which is due to the inefficiency in ESBL detection. In many hospital settings there are no proper testing protocols for the testing of ESBL. This insufficiency has lead to the increased spread of resistant strains. The laboratory settings should become capable enough in order to detect ESBL isolates on routine basis so that proper therapy can be given to avoid overuse of antibiotics.^7^

Phenotypic detection of ESBLs can be done by direct screening of clinical samples or screening by disc diffusion method. Double disc synergy test (DDST) and Combined disc tests (CDT) are commonly used methods.^8^ There are only few studies published in Pakistan about the dissemination of ESBL-producing *E. coli* in different environments: healthy individuals, patients, sewage sludge, cattle, chickens and raw meat. Our study aimed to determine the frequency and antimicrobial profile of ESBL-producing isolates of *E. coli* in different environments. The study will also focus on the techniques used to characterise these superbugs from animals and birds faeces.

## METHODS

This cross-sectional study was conducted at The Children’s Hospital and The Institute of Child Health, Lahore from July to December 2015. The study was ethically approved by the ethical committee of The Children’s Hospital and The Institute of Child Health, Lahore. *E. coli* were isolated from faecal specimens of healthy individuals, patients, sewage sludge, cattle, chickens and raw meat. All the specimens were inoculated on the MacConkey agar medium containing 2mg/L cefotaxime. The specimens from healthy individuals were directly inoculated on the media. The floor samples (10g) of faeces from animals were homogenized in 5mL of peptone water. Aliquots of 0.1 mL were inoculated on the media. The raw meat was collected from various butcher shops of Lahore city. A swab was moistened with the peptone water and was rubbed on the meat surface firmly. Another dry swab was taken and rubbed over the same surface. Both the swabs were introduced into the bottle containing 3-4 glass beads and an appropriate known volume of diluents (0.1% peptone, 0.9% NaCl) and were shaken vigorously. Aliquots were inoculated on the above mentioned media. The sludge samples (1 mL) were inoculated in 2 mL of Brain Heart Infusion (BHI) broth which contained cefotaxime (2 mg/L) and then the sample was incubated at 37°C for two hours under aerobic conditions. After the pre-enrichment procedure, 2 mL from each tube was inoculated in 10 mL of Brain Heart Infusion-Cefotaxime (BHI-CTX) broth. Tubes were further incubated for 18 hours at 37°C under anaerobic conditions to avoid overgrowth of aerobic Gram-negative bacteria. Each culture was diluted 10 fold with Ringer solution and 0.1 mL of each dilution was inoculated on MacConkey agar containing 2mg/L cefotaxime. All the plates were incubated at 37°C for 48 hours.^9^ Identification of bacteria was performed by using colony morphology, routine biochemical test and API 10S.

Detection of ESBL production was confirmed by the Double Disc Synergy Test (DDST). In this process disc containing co-amoxiclav was placed at the centre of the plate and cephalosporins were applied edge to edge at the distance of 15mm from the centre disc. Positive results were indicated when the inhibition zones around any of the cephalosporin discs augmented in the direction of the disc containing clavulanic acid.^10^

Antimicrobial sensitivity testing was performed by using modified Kirby-Bauer disc diffusion technique. An inoculum was prepared according to the McFarland 0.5 turbidity standard and streaked with a swab on Mueller Hinton agar plate. Various antibiotic discs such as amikacin, amoxicillin, cefixime, cefotaxime, ceftazidime, ceftriaxone, cefuroxime, chloramphenicol, ciprofloxacin, fosfomycin, moxifloxacin, cefoperazone-sulbactam, piperacillin-tazobactam, imipenem, meropenem, were used for the antimicrobial susceptibility testing and results were observed after 16-18 hours of incubation at 37°C.^11^ American type culture collection (ATCC) strains were used as controls in this study. *Klebsiella pneumoniae* ATCC, 700603 (ESBL-producing isolate) and *E. coli* ATCC, 25922 (non-ESBL) were used as positive and negative controls for ESBL production, respectively.^12^

## RESULTS

A total number of 122 samples collected from various sources such as hospitalized patients (n=28), healthy individuals (n=30), cattle faeces (n=15), sewage sludge (n=7), raw meat (n=20) and chicken faeces (n=22) were processed for microbiological analysis. Among these samples initial screening showed positivity in 77 (63.0%) samples. The confirmation of ESBL-producing *E*. *coli* was done using DDST which showed 74 (60.0%) isolates as ESBL-producers and three isolates of AmpC beta-lactamases producers which were excluded from study. The frequency of ESBL-producing *E. coli* was found to be 15 (53.0%) in patients, 17 (57.0%) in healthy individuals, 10 (66.0%) in cattle faeces, 5 (71.0%) in sewage sludge, 14 (70.0%) in raw meat and 13 (59.0%) in chicken faeces (Table-I).

**Table-I:** Frequency of ESBL-producing *E. coli* from various sources.

*Source*	*No. of samples*	*ESBL positive E. coli n (%)*	*ESBL negative E. coli n (%)*
Patients	28	15 (53.0)	13 (47.0)
Healthy individuals	30	17 (57.0)	13 (43.0)
Cattle faeces	15	10 (66.0)	5 (34.0)
Sewage sludge	7	5 (71.0)	2 (29.0)
Raw meat	20	14 (70.0)	6 (30.0)
Chicken faeces	22	13 (59.0)	9 (41.0)

Total	122	74 (60.0)	48 (40.0)

The antimicrobial sensitivity pattern of ESBL-producing *E. coli* was observed against 13 different antibiotics. All of the confirmed ESBL-producing isolates were resistant to ceftazidime, ceftriaxone, cefotaxime, cefuroxime and co-amoxiclav. There were 30 (40.0%) isolates which showed resistance to moxifloxacin and 24 (33.0%) to ciprofloxacin. There were 7 (10.0%) isolates resistant to meropenem. None of the isolates showed resistance to cefoperazone-sulbactam, imipenem, piperacillin-tazobactam and amikacin (Table-II).

**Table-II:** Antimicrobial sensitivity of ESBL-producing *E. coli*.

*Antibiotics*	*Sensitive n (%)*	*Intermediate n (%)*	*Resistant n (%)*
Amikacin (30µg)	68 (92.0)	0 (0.0)	6 (8.0)
Moxifloxacin (5µg)	44 (60.0)	0 (0.0)	30 (40.0)
Co-amoxiclav (20/10µg)	0 (0.0)	0 (0.0)	74 (100.0)
Ceftazidime (30µg)	0 (0.0)	0 (0.0)	74 (100.0)
Ceftriaxone (30µg)	0 (0.0)	0 (0.0)	74 (100.0)
Cefotaxime (30µg)	0 (0.0)	0 (0.0)	74 (100.0)
Cefuroxime (30µg)	0 (0.0)	0 (0.0)	74 (100.0)
Cefixime (5µg)	0 (0.0)	0 (0.0)	74 (100.0)
Cefoperazone-sulbactam (105µg)	74 (100.0)	0 (0.0)	0 (0.0)
Ciprofloxacin (5µg)	50 (67.0)	0 (0.0)	24 (33.0)
Piperacilin/tazobactam (100/10µg)	74 (100.0)	0 (0.0)	0 (0.0)
Meropenem (10µg)	67 (90.0)	0 (0.0)	7 (10.0)
Imipenem (10µg)	74 (100.0)	0 (0.0)	0 (0.0)

## DISCUSSION

The present study aimed to evaluate the frequency of ESBL-producing *E. coli* in different environments which could be an important source of dissemination. In our study the frequency of ESBL-producing *E. coli* in patients was 15 (53.0%) and 17 (57.0%) among healthy individuals. In Spain a research was done on the detection of ESBL-producing strains in different environment. The frequency of ESBL-producing *E. coli* was 6.6% in the patients’ stool samples.^9^ Another research done in medical University of Graz, Austria reported 4% increased prevalence of ESBL-producing *E. coli* from 2000 to 2009.^13^ ESBL-producing *E. coli* had 41% prevalence rate from a tertiary care hospital of Pakistan.^14^ A study conducted in Thailand on faecal specimens from the healthy individuals reported that majority of the isolates were ESBL-producing *E. coli* (85.1%).^15^ ESBL-producing *E. coli* were the predominant isolate from the three rural provinces of Thailand where the prevalence came out to be 90%, 90%, and 89%, respectively.^16^ In 2012, healthy individuals who visited the Parisian checkup centre participated and provided the stool samples. Out of 345 subjects, 21 (6.0%) were the ESBL-producing *E*. *coli*.^17^ The frequency of ESBL-producing *E. coli* in these studies is variable. The prevalence of ESBL-producing *E. coli* was low in developed countries like Spain and Austria while it was high in developing countries like Pakistan and Thailand.

In the present study the prevalence of ESBL-producing *E. coli* in sludge samples was 5 (71.0%). A study conducted for the detection of ESBL-producing *Enterobacteriaceae* from various environments in Spain reported 100% prevalence of ESBL-producing *E. coli* from the 5 samples of sewage sludge. All the sludge samples were taken from the treatment plants of influent raw urban sewage.^9^ Only 0.5% ESBL-producing *E. coli* were isolated from sludge samples from 2000 to 2009 in a study conducted in Medical university of Graz, Austria. The samples were taken only from those treatment plants which did not receive any wastewater from hospitals.^13^ ESBL-producing *E. coli* in cattle faecal samples were 10 (66.0%) in our study. A research was done in Mecklenburg-Western Pomerania, Germany detected 54.5% ESBL-producing *E. coli* in livestock cattle.^18^ Another study conducted on Bavarian dairy and beef cattle farms reported the prevalence of 38.0% ESBL-producing *E. coli*.^19^ These results are similar to the results of our study.

The prevalence of ESBL-producing *E. coli* in chicken faecal samples in ourstudy was 13 (59.0%). A research done in Spain reported 100% prevalence of ESBL-producing isolates in chicken floor faecal samples.^8^ In another research conducted in Germany, 72.5% of prevalence rate of ESBL-producing *E. coli* was found in chicken faecal floor samples.^20^ In current study the *E. coli* were isolated from all of the 20 samples of raw meat and there were 14 (70%) ESBL-producing *E. coli*. The samples of raw meat were taken right after their collection from the butcher shops. They were not processed with any cleaning agent before the culturing so that we could see if the animal was slaughtered and treated under hygienic conditions or not. High prevalence of *E. coli* depicts the unhygienic ways of handling the meat. A research conducted in Netherland for the detection of ESBL-producing *E. coli* reported prevalence of 79.8% in chicken raw meat samples.^21^ Another study conducted in Germany reported the prevalence of 88.6% in raw meat samples.^20^ The above results of different studies are in accordance with our study result.

All the ESBL-producing *E. coli* was resistant cephalosporins and co-amoxiclav in our study. A study from Germany also reported high resistance to ESBL-producing *E. coli* to the cefixime, cefuroxime, cefotaxime, ceftazidime and ceftriaxone.^20^ Majority of the ESBL-producing *E. coli* in our study presented with good sensitivity against cefoperazone-sulbactam, imipenem, piperacillin-tazobactam and amikacin. A research done on Parisian checkup centre showed the sensitivity pattern in which none of the ESBL-producing *E. coli* isolate was resistant to piperacillin-tazobactam, imipenem or amikacin.^17^ The prevalence of ESBL-producing *E. coli* was notable in all the environments studied in our research, suggesting a global expansion of these enzymes to make them superbugs. This prevalence is likely to increase among humans worldwide in the future due environmental dissemination.

ESBL producing strains tend to show resistance towards cephalosporins which lead towards the gene pool of strains with high level of resistance in the environment. The environment in this way has become a great reservoir of ESBL strains. This situation has dragged our condition towards increased load of antibiotics, poor clinical outcome and limited therapeutic options.

### Authors’ Contribution

**RS:** Conceived the idea, monitored data collection, & drafted the paper.

**HE:** Data organization, data compilation, proof reading

**AZ:** Designed the study, performed data analysis, & assisted in writing

**SY:** Reviewed the literature, designed the study tool, and reviewed manuscript.

**AWR:** Takes the responsibility and is accountable for all aspects of the work in ensuring that questions related to the accuracy or integrity of any part of the work are appropriately investigated and resolved.
